# A Quest for Initiating Cells of Head and Neck Cancer and Their Treatment

**DOI:** 10.3390/cancers2031528

**Published:** 2010-07-27

**Authors:** Chao Chen, Beate Köberle, Andreas M. Kaufmann, Andreas E. Albers

**Affiliations:** 1 Department of Otolaryngology and Head and Neck Surgery Charité-Universitätsmedizin Berlin, Campus Benjamin Franklin, Hindenburgdamm 30, 12200 Berlin, Germany; 2 Department of Head and Neck Surgery, Zhejiang Cancer Hospital, China; E-Mail: lancet2000@msn.com; 3 Institute of Toxicology, University Medical Center, Mainz, Germany; E-Mail: koeberle@uni-mainz.de; 4 Clinic for Gynecology, Charité-Universitätsmedizin Berlin, Berlin, Germany; E-Mail: andreas.kaufmann@charite.de

**Keywords:** metastasis, stemness, ALDH1, Sox2, Nanog, Oct3/4, human papillomavirus, immunotherapy, chemoresistance, radioresistance, epithelial mesenchymal transition

## Abstract

The biology of head and neck squamous cell carcinomas (HNSCC) and other cancers have been related to cancer stem-like cells (CSC). Specific markers, which vary considerably depending on tumor type or tissue of origin, characterize CSC. CSC are cancer initiating, sustaining and mostly quiescent. Compared to bulk tumors, CSC are less sensitive to chemo- and radiotherapy and may have low immunogenicity. Therapeutic targeting of CSC may improve clinical outcome. HNSCC has two main etiologies: human papillomavirus, a virus infecting epithelial stem cells, and tobacco and alcohol abuse. Here, current knowledge of HNSCC-CSC biology is reviewed and parallels to CSC of other origin are drawn where necessary for a comprehensive picture.

## 1. Introduction

Head and neck squamous cell carcinomas (HNSCC) are the sixth most prevalent type of malignancy worldwide. Despite advances in therapy, which have improved quality of life, survival rates have remained static over the past decades. Mortality from this disease remains high due to the development of distant metastases and the emergence of eventually inoperable local and regional recurrences that have low responsiveness to radiation- or chemotherapy. Therefore, despite significant improvements in surgery, radiation- and chemotherapy, long-term survival rates in patients with advanced stage HNSCC have not significantly increased in the past 30 years [1,2,3]. Even in the case of stage I disease, where 90% of patients can be cured, 10% relapse with fatal outcome. In more advanced stages, the subset of patients who fail to respond to therapy or suffer from recurrences increases, for so far unknown reasons. It is, therefore, desirable to develop a deeper understanding of the biology of this disease to adapt current therapeutic strategies and to develop therapies that are more effective.

Evidence has recently been accumulating to support the hypothesis that solid tumors contain a small subpopulation of cells called cancer stem-like cells (CSC), which exhibit self-renewing capacities and are responsible for tumor maintenance and metastasis [4] and possibly for resistance towards chemotherapy and radiation therapy. The cancer stem cell theory [5] has prompted a re-examination of current therapeutic concepts aiming at developing therapeutics specifically targeting CSC, which might synergize with treatment modalities directed at cancer bulk populations to improve clinical outcome. 

In this context, it is also noteworthy that HNSCC has at least two distinct etiologies. In most instances, HNSCC is either caused by the spontaneous accumulation of multiple genetic alterations modulated by genetic pre-disposition and chronic inflammation, enhanced by environmental influences such as tobacco and alcohol abuse, or by infection with oncogenic human papillomavirus (HPV). Carcinogens are regarded as the most important factors. Thus, two main etiologies can be defined: tumors induced by toxic substances and tumors induced by the activity of the viral oncogenes of HPV. Both etiologies involve a multistep process and result in alterations affecting two large groups of genes: oncogenes and tumor suppressor genes. HPV-associated HNSCC defines a distinct subgroup. More than 100 human papillomavirus (HPV) subtypes are known to date. Of these, 15 have been shown to be oncogenic in humans. HPV type 16 and 18 seem to play the major role in the etiology of HPV-associated HNSCC, particularly those arising in the oropharynx [6,7]. HPV association has been detected in 20% to 30% of tumors located in all head and neck anatomic subsites and in about 50% of tonsil squamous cancers. For laryngeal cancer, the role of HPV is less clear. Data on the prevalence of high-risk HPV-associated HNSCC vary and multicenter studies have not yet been performed [8,9]. 

It is attractive to speculate that HPV, which primarily infects basal cells in the epithelium, indeed infects epithelial stem cells that are transformed to become cancer initiating cells [10]. This concept suits the highly regulated replication and propagation strategy of these viruses.

The lower rate of carcinogenic risk factors and p53 mutations and a younger patient population suggests that factors, currently unknown, are associated with viral entry, propagation/transformation, and immune evasion in HPV-associated HNSCC patients [7,11]. Failure to clear HPV infection leaves host cells under the influence of the viral oncogenes. Persistently infected persons can develop clinically or histologically recognizable precancers that can persist and may develop over time into invasive cancer. These oncogenes are vital to the tumor cell survival and proliferation, and therefore provide a suitable target for anti-tumor vaccination.

Therefore, with these two different etiologies, different treatment options according to the genesis of their malignancy may be developed for future patients. 

This review focuses on the description of known and potential markers for CSC in HNSCC and their potential use for specifically targeting these cells. In order to give a more comprehensive view to this complex topic, parallels to other cancers were drawn and comparisons were made when data for HNSCC were scarce or not yet available. Advances in the research of other cancers with potential translational relevance for HNSCC are briefly addressed. 

## 2. Definition of Cancer Stem Cell-Like Cells

CSC are viewed as the result of the oncogenic process. According to the CSC model, the phenotypic diversity of cells that form a tumor and its metastases derive from CSC and are organized hierarchically [12,13]. It is assumed that, in theory, one CSC can completely regenerate the tumor it has been taken from. Regeneration-rates in transplantation studies have shown that tumor re-initiation is variable, ranging from as low as 20 to 10^7^ cells, depending on the markers used [12,14]. The tumor itself consists of at least two subpopulations: a smaller CSC population and a bulk population of non-tumorigenic cancer cells that have differentiated from CSC and have lost their self-renewing capacity. The concept of this model has been developed in analogy to the renewal of adult tissues like blood that regenerates from a pool of stem cells [15]. 

The first experimental evidence for the existence of CSC came from a subpopulation of acute myeloid leukemia that comprised 0.01–1% of the total population and that could induce leukemia when transplanted into immunodeficient mice [16,17]. The self-renewal properties of CSC are thus the real driving force behind tumor-growth.

A proof of this model has been provided by demonstrating that selective killing of CSC can inhibit tumor growth [18]. Like physiologic tissues, cancers are composed of heterogeneous cell populations [19] that exhibit distinct morphologic and functional phenotypes [20,21,22,23].

CSC combine four properties that also define them: (a) to initiate a malignant tumor and to drive neoplastic proliferation; (b) to recreate the full phenotype of the parent tumor when being transplanted; (c) the expression of a distinctive repertoire of biomarkers compared to the non-CSC tumor cells, and (d) to recreate itself (reviewed in [18,24]). These criteria rely on the thorough establishment and further development of robust criteria, unbiased by experimental procedures to identify and isolate CSC [25]. 

It still remains unclear whether a cancer stem cell is the direct progeny of a mutated stem cell or of more mature cells that reacquire stem cell properties during tumorigenesis (and thereby contradicting somehow the theory of hierarchical order of tissues) [26]. CSC share important properties with normal tissue stem cells (TSC), including the capacity for self-renewal and the expression of stemness factors. Asymmetric divisions of TSC result in hierarchically organized, irreversibly differentiated tissues with physiologic functions that are under homeostatic control. The result of asymmetric divisions of CSC is a hierarchically organized tumor with a most likely stochastic differentiation that may be reversible in part and disorganized tumor tissue architecture. CSC have a relatively high division rate compared to TSC. TSC have the requirement for a specific niche. Whether CSC also reside in a specific niche is unknown. It is also currently unknown whether normal stem cells are susceptible to cancer-causing mutations that directly give rise to CSC. HPV-related HNSCC might be an exception, since it has been shown that HPV infects the basal layer of the mucosa, a stem cell compartment from which the mucosa is being regenerated.

## 3. Dichotomy between Hierarchical and Stochastical Tumor Models?

HNSCC, as other solid tumors, is histologically a highly heterogeneous disease. It consists of the cancer cells that can be subtyped and grouped into populations with stem cell characteristics and more differentiated cell types that possibly resemble a developmental hierarchy in analogy to normal tissues. Attempts to explain the genesis of diversity within a tumor have been made. Currently two competing models exist. The hierarchical model describes a differentiation-like diversification from an initially monoclonal tumor cell lineage. The stochastic model uses events like spontaneous shifts in cell phenotypes to explain heterogeneity.

In addition to the cancer cells, stromal cells and inflammatory cells are found in the tumor bed. Especially in HNSCC, heterogeneity can be well explained by the constant exposure of the oral mucosa to mutagenic agents contained in tobacco products. This exposure results in multiple genetic changes of various degrees in the whole aero-digestive tract, until in one area, a point of no return is reached and a pre-neoplastic field develops. This field is of monoclonal origin and expands non-invasively. Clonal divergence and selection within the field leads to the development of cancer [27]. The phenomenon of tumor evolution by accumulation of stepwise genetic alterations was termed field-cancerization. According to this theory, HNSCC can arise synchronously or metachronously at different sites. 

**Figure 1:**
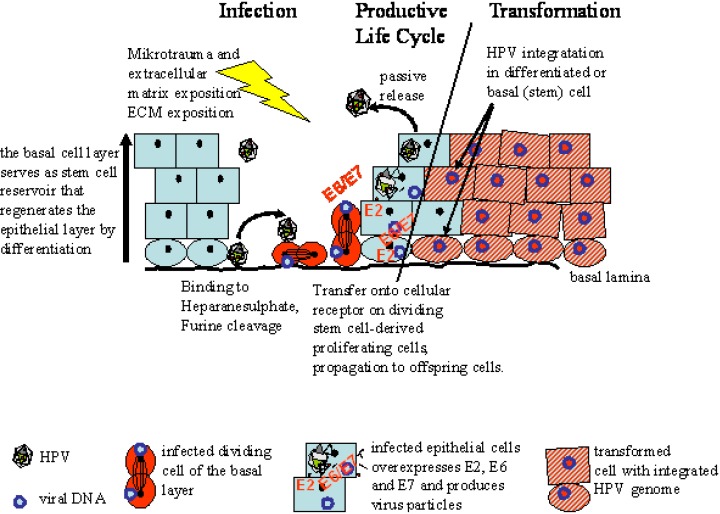
The HPV life cycle involves epithelial stem cell-like cells. Through micro-injury, HPV reaches the basal compartment of the epithelium and binds to extracellular matrix components of the basal lamina. Upon contact to dividing cells, the virus infects these cells and may become quiescent together with the reacquired stem cell phenotype. This allows prolonged persistence of viral infection and fixation of the infection in the epithelial stem cell. This may, in part, also be balanced by the viral oncogenes E6, E7 and the transcription repressor E2. When epithelial cells differentiate, the viral genetic program is switched and structural proteins are induced. During tumor progression integration of the viral genome into the host cell genome occurs at a random position.

In the case of HPV-induced tumors, these processes can be potentially followed using HPV DNA sequences as a tag of those cells prone to immortalization, tumor progression, and metastasis [28]. HPV infection will form a premalignant field of infected cells (intraepithelial neoplastic lesion) with an extended life span and reentering of aberrant cell cycles. These cellular fields have a high probability of acquiring more genetic changes that ultimately may give rise to immortalized and transformed cells further progressing to cancer. HPV has evolved and adapted to infect the stem cell of the epithelium (Figure 1). 

This stem cell-like cell is located in the basal layer close to the basal lamina and is quiescent. Successful infection by HPV therefore requires breaches or micro-injuries of the epithelium, so that the viruses can enter and reach the basal lamina. HPV can attach to the proteoglycans of the basal lamina, which represents a reservoir for infectious particles [29]. Mircoabrasions and injuries will be closed by activation and proliferation of quiescent (stem)-cells of the tissue. These cells and this physiological state of proliferating undifferentiated pluripotent cells are most probably the target for HPV infection [30]. However, it is not certain that these cells are true pluripotent stem cells. 

Upon infection, the viral DNA is maintained in the progeny of this cell for prolonged periods, maybe as long as this cell clone is present. Only when these HPV-positive stem cells divide again and finally differentiate to become proliferating suprabasal cells that build up the epithelium, the HPV switches gene expression to produce structural proteins that form the viral shell. Finally, viral particles are released passively with descaling cells. This life cycle is dependent on a close interaction of viral and cellular genes and possibly genes regulating the stem cell character of the basal epithelial cell. In terms of field cancerization, HPV initially produces a premalignant lesion of heterogeneous cells with different states of the viral genome. With persistence of the infection and progression towards malignant transformation, the viral genome integrates into the host cell genome at a random position. This integration event, henceforth, will identify this cell’s progeny. This represents a genetic tag of the cell clone, and it turns out that tumors arise as monoclonal expansion of a single cell [31]. It is currently not known if this happens in a differentiated epithelial cell that subsequently will give rise to new cancer stem cells or if this has to happen in a basal (stem) cell. It is attractive to speculate that in the resulting tumors, CSC will be formed from the HPV-tagged differentiated cells and thus are derived from the non-stem cell compartment of the epithelium. Anyhow, this integration event supports the development of a malignant phenotype. Loss or inhibition of viral transcription leads to apoptosis. Therefore, all cells of the tumor, the bulk tumor cells, the metastasizing, and the CSC, are tagged with HPV as a unique marker. Of course, over time and during progression to more malignant phenotypes additional genetic and epigenetic changes occur leading to heterogenization of the tumor mass. The clonal origin, however, will be maintained as documented by the HPV integration site. In comparison to spontaneously arising HNSCC that do not carry a conclusive marker, the “HPV-tagged” tumors may offer an ideal opportunity to investigate and discriminate CSC and bulk tumor cells and their origin and fate.

Many of the biological phenomena occurring in HNSCC can be well explained by this stochastic model. For example, due to the large size of the preneoplastic fields expanding beyond typical surgical margins, local recurrences or secondary cancers can be explained. The most obvious limitation of the evolutionary progression model is to explain the cellular heterogeneity observed in tumor nests, which, in contrast, can be well explained by the hierarchical CSC model. The CSC theory of solid tumors is supported by the observation of cancer cells in close proximity that have different biological behavior (e.g., to metastasize). This can be explained rather by differentiation than evolution [32]. Whether this is also the case in HNSCC remains to be shown. Importantly, the metastases, putatively arising from single or few metastasizing tumor cells, also show heterogeneity, although exposure to DNA-damaging agents similar to the primary tumor is absent. 

The difference of CSC cells and tumor bulk cells can be demonstrated by their biological behavior, which has been used to characterize or identify CSC candidates. 

Therefore, the following questions have to be asked: 

What can the CSC-theory add to the current concept? 

Where do the evolutionary/stochastical model and the hierarchical CSC model meet and where do they contradict? 

What are the limitations to the theories? 

Currently these questions cannot be answered to a satisfying degree for HNSCC. Analogies to other solid tumors have to be drawn until more knowledge is accessible. Finally, all information should be integrated into a comprehensive model for HNSCC. 

## 4. Assays for CSC Research

### 4.1. The Xenotransplant Tumor Initiation Assay

The xenotransplant tumor-initiation assay is probably the most important assay in CSC research. Xenotransplantation has been widely used to investigate tumorigenicity and metastatic capacity of human tumor specimens and cell lines. Generally, a piece of tumor tissue or a cell suspension is transplanted subcutaneously or orthotopically and leads to outgrowth in immunocompromised mice. Depending on the tumor line, a large number of transplanted cells are necessary. This is probably due to the low number of tumor initiating cells that are initially present. In contrast, metastasizing cells can be identified by subsequent formation of secondary tumors. However, while the presence of tumor initiating stem cells is a prerequisite for sustained tumor growth and retransplantation, metastasis from solid tumors is an independent characteristic not shared by all tumor lines. Therefore, the metastatic and the tumor stem cell may be different entities in the bulk tumor mass and are not identical in their capabilities of local and disseminated growth, invasion or quiescence. 

The xenotransplatation assay allows for experimental verification of the stemness of a given cancer cell subset. With this experimental approach, tumorigenic cell populations (e.g., from tumor biopsies) that are capable of sustained growth in mice, allowing unlimited retransplantation of human cancer xenografts into mice, could be identified and subsequently characterized. In addition, subpopulations carrying a specific marker can be tested for their stemness with this assay. 

However, a risk for experimental bias in this assay was recently demonstrated in melanoma. The original frequency of CSC in melanoma was observed to be in the order of 1 in 10^5^ cells. This low frequency was found to be an artifact due to residual innate immunity of the recipient mouse. In an improved model with a more profoundly immunodeficient recipient, deficient in T-, B-, NK-cells, and macrophages, 15–25% of cells showed CSC activity [33]. Of note, these results raise the question of truly hierarchical organization of the tumor in melanoma (reviewed in [34,35]). Whether this is also the case in HNSCC remains to be determined. Another possible limitation of these models is the murine microenvironment that may not always be sufficient to reveal CSC or stemness potential, and it has even been argued that species differences alone might account for the selective growth of subpopulations of cells in this assay.

A third limitation of the model is that it relies on separate testing of sorted cell populations. A demonstration of a hierarchical tumor organization is virtually impossible. Also, a conversion of a marker phenotype among cancer subpopulations, as has been reported for the candidate CSC marker CD133 [36], rules out an unambiguous demonstration of hierarchical CSC driven tumor organization. 

Therefore, marker-specific genetic lineage tracking (see below) of cancer subpopulations in competitive tumor development models enhances cell sorting-based xenotransplantation assays and can serve to confirm the existence of tumor hierarchies driven by molecularly defined CSC as was recently shown in melanoma [37].

### 4.2. Genetic Lineage Tracking

Genetic lineage tracking can be used to identify a tumor hierarchy. In principle, this can be achieved by identifying genetic aberrations or epigenetic modifications that occur during gene regulation (recently reviewed in [25]). The specific properties of CSC are reflected by expression patterns that are characteristic for the physiological state of a stem cell. For example, certain transcription factors may be permanently regulated by promoter methylation. However, epigenetic markers are less stable than true genetic events like mutations or viral integration. As described above, viral integration—like HPV sequences that are inserted into the genome of the host cell that eventually progress to become a tumor cell—could also be an adequate tool to follow the fate and lineage of tumor cells [31]. This could be an attractive and alternative tag for certain tumors that carry integrated viral DNA.

Genetic lineage tracking of marker-sorted cancer subpopulations allowed the identification of molecularly defined CSC at the apex of hierarchically organized tumors in human malignant melanoma [37] and colon cancer [38,39] and helped to identify tumor subpopulations of enhanced tumorigenicity in breast cancer and glioma cell lines [40]. All of these reports demonstrate nicely the use of genetic tools for lineage tracking in murine models to provide evidence for a tumor hierarchy. 

### 4.3. Non-Adherent Sphere Formation Assays

Non-adherent sphere formation assays are increasingly being used to evaluate stem cell activity in normal tissue and putative CSC. They rely on the ability of most stem cells of epithelial origin to grow floatage independently in suspension forming clusters of cells on non-adherent surfaces. The neurosphere is the best-studied sphere assay. The central nervous system cells grown on nonadherent surfaces give rise to neurospheres that have the capacity for self-renewal and can generate all of the principal cell types of the brain [41,42]. The capacity for repeated generation of neurospheres from single cells is generally viewed as evidence of self-renewal [43]. Recently, spheroids isolated from gliosarcoma rat cell lines were also shown to possess cancer stem-like cells [44]. There is also emerging evidence that spheroids grown from HNSCC cell lines or even primary tumor are enriched for CSC. 

To generate spheres, single cells were seeded in ultra-low attachment plates at a low density. After five to seven days, representative spheroids formed and could be collected for following experiments. However, it has to be noted that spheres still consist of heterogeneous populations, which are enriched in CSC but not entirely pure or can be produced from any cell line.

Many of these assays test for abilities of the CSC that relate to their self-renewal and metastasis capacity. For metastasis, the phenomenon of transdifferentiation as observed in an epithelial-to-mesenchymal transition process is important.

## 5. The Role of Epithelial-Mesenchymal Transition

Epithelial-mesenchymal transition (EMT) is a key step during embryogenesis, but recent evidence also suggests that genetic programs relevant for EMT are also transiently activated in epithelial cancers playing a role in cancer progression, through which transformed epithelial cells invade tissues and metastasize. 

Although the EMT program is necessary for normal development, the aberrant activation of EMT contributes to various pathologic conditions, including fibrosis and carcinoma progression [45,46]. During EMT, epithelial cells break down cell-cell and cell-extracellular matrix connections and migrate to other locations in the body [47]. During cancer progression, EMT seems to provide cancer cells with the capacity to infiltrate the surrounding tissue and ultimately metastasize to distant sites [48].

**Figure 2:**
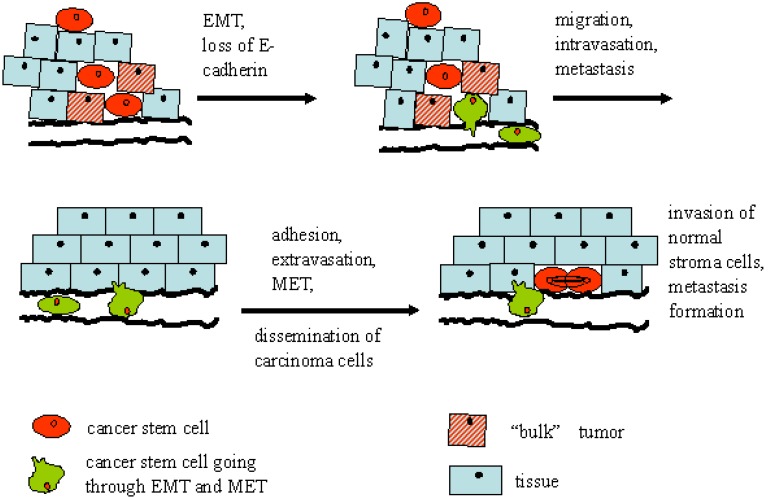
CSC potential for EMT during metastasis formation. Metastasis is the main cause of death in cancer patients. However, not every cell in a tumor has the ability to metastasize to other organs. In the tumor bed, CSC reside in niches (hatched cells) that support the quiescent phenotype. Some CSC may acquire the potential to transdifferentiate from an epithelial to a mesenchymal cell phenotype (EMT). EMT, or loss of differentiation, is frequently observed in many epithelial tumors at the invading edge of the tumor. CSC lose E-cadherin expression and contact to adjacent cells. As a first step of metastasis, they acquire the potential to migrate through extracellular matrices and evade into lymph or blood vessels. In the target tissue of metastasis CSC extravasate and invade the tissue. The CSC transit back to an epithelial phenotype (MET), re-express E-cadherin and proliferate to build up a secondary tumor (metastasis). CSC that can transit through EMT and MET are often called metastatic CSC.

Once the migrating mesenchymal cells have reached their destination, they can undergo a reverse EMT, a mesenchymal-epithelial transition (MET) (Figure 2) [47]. The EMT and the reverse process, MET, play important roles in embryogenesis [49,50,51] and wound healing [52]. It has been shown that non-EMT cells were unable to metastasize without the action of EMT cells in animal experiments, suggesting that EMT- or EMT-like processes may be required for metastasis [53]. Accumulating evidence also shows that EMT plays a key role in cancer chemoresistance [54,55,56]. Recently, it has been reported that the induction of EMT in differentiated immortalized human mammary epithelial cells by either compulsory expression of stem cell transcription regulating factors Snail or Twist or exposure to TGF-β1 caused the cells to acquire the CD44^high^/CD24^low^ stem cell profile [57]. The authors also showed that putative CD44^high^/CD24^low^ breast cancer stem cells isolated from neoplastic human breast tissues expressed high levels of mRNAs encoding EMT-associated markers. Another group reported that cells possessing both stem cell and tumorigenic characteristics of ‘‘cancer stem cells’’ can be derived from human mammary epithelial cells following the activation of the Ras-MAPK pathway. The activated cells expressed low or undetectable levels of the epithelial markers E-cadherin and beta-catenin and high levels of the mesenchymal markers vimentin and fibronectin, suggesting that they underwent an EMT [58].

For HNSCC, it was recently shown that overexpression of the receptor tyrosine kinase TrkB resulted in altered expression of molecular mediators of EMT, including downregulation of E-cadherin and upregulation of Twist. This observation was confirmed in a mouse model by showing that downregulation of TrkB suppressed tumor growth. These results directly implicate TrkB in EMT and the invasive behavior of HNSCC, and correlate with the *in vivo* overexpression of TrkB in human HNSCC [59].

## 6. Potential Markers of CSC in Head and Neck Cancer and Their Relevance

According to the CSC model, tumors consist of heterogeneous cell populations in distinct phenotypic and functional states that are hierarchically organized. If these cancer cell populations, as it seems, respond differently to cancer therapy, it is desirable to identify and purify each population to investigate possible susceptibilities and to understand their possibly unique biology. Much effort has, therefore, been made to identify candidate markers that are either useful for fractionation of cell populations for further investigation, or as target-structures for specific therapies. These markers could be cell-surface markers or molecules involved in specific metabolic or signaling pathways. 

To date, no universal CSC marker for solid tumors has been identified. The future will show whether such a marker exists at all. It is possible that CSC markers are tumor specific for the tissue of origin and the niche from where the tumor is growing. Table 1 gives a summary of currently used candidate markers of HNSCC and a number of other solid tumors, which are reviewed in detail elsewhere [4]. 

**Figure 3:**
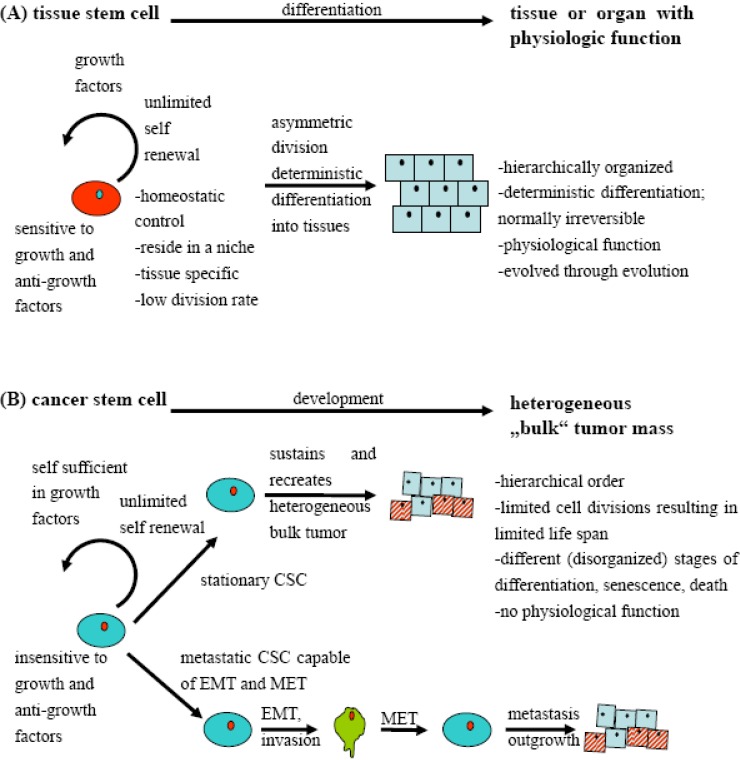
Comparison of (**A**) the development of tissue/organs from stem cells, and (**B**) the development of a heterogeneous tumor mass from cancer stem cells. Normal somatic stem cells underlie a homeostatic control and divide or differentiate in an ordered fashion. CSC, although sharing characteristics with normal tissue stem cells, support unlimited growth by replenishing with juvenile tumor cells. They enable the process of metastasis of the tumor by epithelial-mesenchymal transition (EMT) and the reverse process termed mesenchymal-epthelial transition (MET) that allows dissemination.

CSC share many characteristics with normal stem cells, with self-renewal and differentiation being the most important. Although the molecular mechanisms of these signal transduction pathways might be the same, their regulation in CSC may be deregulated and participate in tumor growth (Figure 3).

Comparative studies of normal stem cells and CSC from the same tissues showed that, for instance, the signaling pathways of Bmi1 and Wnt have similar effects in self-renewal, suggesting that common molecular pathways regulate both populations.

Initially, CD44^+^CD24^−/low^ cells were proposed to exhibit CSC properties and are regarded as CSC for breast cancer [83]. Subsequently, CD133 was found to mark CSC in brain tumors [72], colorectal carcinoma [73] and pancreatic carcinoma [84]. In head and neck cancer, Prince *et al.* were the first to demonstrate that the population of HNSCC cells possess the properties of CSC [66], but a relatively high number of CD44^+^ cancer cells (>5,000 cells) are needed to generate new tumors in immunodeficient mice. Moreover, one group reported that CD44s and CD44v6 expression does not distinguish normal from benign or malignant epithelia of the head and neck. CD44s and CD44v6 were abundantly present in the great majority of cells in head and neck tissues, including carcinomas [64]. Thus, identification of more specific CSC markers for HNSCC is still needed. Recently, high aldehyde dehydrogenase 1 (ALDH1, also known as ALDH1A1) activity was shown to identify the CSC in breast cancer, lung cancer, hepatoma, head and neck, and colon cancer [14,60,61,62,63]. However, in breast cancer the ALDH1^+^ population shows a surprisingly small overlap with the previously described CD44^+^CD24^−/low^ phenotype (0.1–1.2%). The cells bearing both phenotypes appeared to be highly enriched in tumorigenicity, being able to generate tumors from as few as 20 cells [14]. It remains to be determined if there is also a small overlap of stem cell markers in HNSCC.

Expression of CSC markers like ALDH1 can directly effect detoxifying molecules in a cell and may explain increased resistance of CSC to cytotoxic reagents.

**Table 1:** Cancer stem cell markers of HNSCC and other solid tumors. To date, no generic marker for all CSC has been described. CSC of different tumor entities have distinct markers that do not always overlap. The future will reveal whether a universal CSC marker exists or if CSC are tumor-type specific, depending on the origin of each type of CSC.

CSC marker	Origin	Function/physiological role	Ref.
CD44^+^/CD24^−/low^ (ALDH1^+^)	HNSCC, breast, lung, hepatoma	CD44: A cell-surface glycoprotein involved in cell-cell interaction, cell migration, and adhesion with multiple isoforms that has pleiotropic roles in signaling and homing. The standard form CD44H exhibits a high affinity for hyaluronate; CD44V confers metastatic properties. Several CD44 splice variants are known as being associated with cell transformation. The standard form of CD44 (CD44s) was shown to be part of the signature of cancer stem cells (CSC) in colon, breast, and in head and neck squamous cell carcinomas (HNSCC). This is somewhat in contradiction to previous reports on the expression of CD44s in HNSCC. CD24: A cell adhesion molecule expressed at the surface of most B cells and differentiating neuroblasts.	[14,60,61,62,63,64,65,66]
CD44^+^ Lineage^-^	HNSCC (controversial)		[64,66]
CD44^+^/EpCAM^hi^	Colon	EpCAM: Homophillic Ca2+-independent cell adhesion molecule expressed on the basolateral surfaces of most epithelial cells.	[67]
CD44^+^/CD24^−^/ESA^+^	Pancreas		[68]
ALDH1^+^	HNSCC, Breast	ALDH1: A member of the ubiquitous aldehyde dehydrogenase (ALDH) family of cytosolic enzymes that catalyse the oxidation of aliphatic and aromatic aldehydes to carboxylic acids. ALDH1 has a role in the conversion of retinol to retinoic acid, which is important for proliferation, differentiation and survival. Furthermore, ALDH1 enzymatic activity has been identified as responsible for the resistance of progenitor cells to chemotherapeutic agents.	[14,63,69,70]
CD133^+^	HNSCC, CNS, colon, Ewing’s sarkoma, pancreas, lung, liver	CD133 (Promenin 1): A pentaspan transmembrane glycoprotein domain expressed in several stem cell populations and cancers. Possible role in the organization of plasma membrane topology.Expressed on CD34^+^ stem and progenitor cells in fetal liver, endothelial precursors, fetal neural stem cells, and developing epithelium. CD133 has been detected by its glycosylated epitope in the majority of studies. Thus, CD133 may be a more reliable cancer stem cell marker	[36,71,72,73,74,75,76]
Side population (Hoechst dye)	Mesenchymal	Side population (descriptive term derived from flow-cytometry experiments): Phenotype due to the Hoechst33342 efflux pump present on the plasma membrane in diverse cell types. Activity conferred by the ABC transporter ABCG2.	[77]
ABCG5^+^	Melanoma	ABCG5^+^: Member of the ATP binding cassette family, involved in transport of sterol and other lipids. ABCG2 (also known as breast cancer resistance protein) is a multi-drug transporter (see Hoechst SP below). ABCG5 confers doxorubicin resistance.	[37]
Snail	HNSCC	Snail: Transcriptional repressor upregulated in EMT and modulated by IL-1beta. Regulates COX-2-dependent E-cadherin expression	[63,78]
Twist	HNSCC	Twist: Transcription factor during embryonic development and has recently been found to promote the EMT phenomenon seen during the initial steps of tumor metastasis in various cancers. It regulates the expression of several genes involved in differentiation, adhesion and proliferation.	[59,79,80]
Oct-4	HNSCC, embryonic stem cells (ES), many others	Oct-4: Transcription factor expressed in pluripotent embryonic stem (ES) and germ cells. Oct-4 mRNA is normally found in totipotent and pluripotent stem cells of embryos. Knocking out the Oct-4 gene in mice causes early lethality due to the lack of inner cell mass formation, indicating that Oct-4 has a critical function for self-renewal of ES cells. Oct-4 activates transcription via octamer motifs, and Oct-4 binding sites have been found in various genes fibroblast growth factor 4 and platelet-derived growth factor α receptor. This suggests that Oct-4 functions as a master switch during differentiation by regulating the pluripotent potentials of the stem cell, and Oct-4 plays a pivotal role in mammalian development.	[71,81]
SOX2	ES, many others	The transcription factor SOX2 is essential for maintaining the pluripotent phenotype of ES cells and is a partner of Oct 3/4 in regulating several ES cell-specific genes. Oct 3/4 and SOX2 interact specifically and bind to a composite regulatory element. Activation of this element maintains Oct 3/4 and SOX2 expression in pluripotent cells.	[82]
Nanog	HNSCC, ES, many others	Nanog, like Sox2 and Oct4, is a transcription factor essential to maintaining the pluripotent ES cell phenotype. Through a cooperative interaction, Sox2 and Oct4 have been described to drive pluripotent-specific expression of a number of genes.	[71,81,82]

## 7. Cancer Stem Cells—adiation and Chemotherapy

In contrast to many other solid cancers, distant metastases in HNSCC are rarely present at diagnosis, but due to improved local control, the incidence of systemic spread is increasing. Patients with recurrent or metastatic disease have a poor prognosis, with median survival rates of 6–10 months [85]. In these instances, chemotherapy, apart from antibody-treatment, remains the only systemic treatment option, which is whenever possible combined with surgery and radiation. Clinically, the use of radiation and high-dose chemotherapy in many instances results in a good initial response of the tumor, if dosage is not limited by co-morbidities of the patients. Unfortunately, the development of recurrences unresponsive to further treatment is frequent and raises the question for the underlying causes. Advances in CSC research may provide some explanation of these phenomena.

In the past few years, studies have begun to investigate the role of CSC for the therapeutic resistance of cancers. In these studies, cell surface markers were used to identify and purify CSC from tumors. It could be shown that the fraction of CSC is enriched in tumor samples or cancer cell cultures after treatment with radiation or chemotherapeutic drugs, and it was therefore proposed that CSC are particularly resistant to radiotherapy and chemotherapy. This resistance might then contribute to treatment failure. Consequently, CSC could represent a novel target for therapeutic treatment.

In a number of studies, evidence for radio- and chemoresistance of CSC has been presented. The mechanisms underlying this resistance are not yet fully elucidated, but are under investigation. With respect to the importance of CSC for chemo-radioresistance in head and neck cancers, HNSCC are relatively under-investigated. Even though the tumor-forming ability of HNSCC-CSC has been reported in a number of studies [65,76], only little is known about the role of CSC for chemo- and radioresistance in HNSCC. Using aldehyde dehydrogenase 1 (ALDH1) as a marker for CSC in HNSCC, Chen *et al.* showed that HNSCC ALDH1^+^ cells were tumorigenic and displayed resistance towards radiotherapy [63]. 

More information about the role of CSC for resistance to conventional cancer therapies is available for other solid tumors and hematological malignancies. Bao *et al.* found that irradiation enriched the subpopulation of cells expressing CD133^+^, a marker for brain cancer stem cells, in glioblastoma tumor specimens as well as xenografts and cell cultures derived from glioma xenografts. The CD133^+^ cells were considerably more radioresistant, which was shown to be at least in part due to elevated DNA damage response and an increase in DNA repair capacity [86]. In a separate study, Liu *et al.* demonstrated that CD133^+^ cells derived from human glioblastoma were resistant to various chemotherapeutic agents, compared to CD133^-^ cells. The evaluation of CD133 in cancer tissue of patients showed a significantly higher expression in recurrent glioblastoma as compared to the respective newly diagnosed tumor [87]. Similarly, CD133 expressing glioblastoma stem cells, isolated from tumor xenografts, showed a marked resistance towards a number of chemotherapeutic drugs including etoposide, cisplatin, and temozolomide [88]. The reason for the observed resistance are not yet clear, but it was assumed that stem cells derived from brain tumors have an altered expression of proteins related to apoptosis rendering them resistant [88]. The role of CSC for chemoresistance has also been studied in established permanent glioblastoma cancer cell lines. CSC were derived from the U87MG glioblastoma cell line using spheroid culturing and investigated for drug resistance. Compared to parental U87MG cells, U87 stem cells possessed a higher drug resistance to anticancer drugs including doxorubicin, etoposide, carboplatin and BCNU and stained positive for multidrug resistance (MDR)1 suggesting that CSC may be resistant to chemotherapy due to increased expression of MDR1 [89]. Ropolo *et al.* isolated CD133 expressing cells from monolayer cultures grown out of glioma tumor specimens or from established permanent glioma cell lines. The CD133^+^ cells showed increased resistance to radiation compared to non-stem glioma cells. Investigation of the mechanisms underlying the resistance phenotype revealed enhanced activation of Chk1 and Chk2 kinases in the CD113^+^ glioma cells compared to CD133^−^ cells, while no increase in DNA repair pathways was observed. DNA repair did not seem to contribute to radioresistance in glioma stem cells, but the phenotype seems to be rather due to enhanced activation of checkpoint proteins [90]. 

Evidence for CSC-associated resistance has also been presented in breast cancer. Phillips *et al.* showed that irradiation increased the fraction of CSC-like cells in breast cancer cell cultures and these CSC-like cells were considerably more resistant towards radiation compared to the corresponding monolayer cultures [91]. Li *et al.* compared the CSC population in paired breast cancer biopsies, which were obtained from patients before and after chemotherapy. They found that the percentage of the highly tumorigenic subpopulation of CD44 expressing cells was increased in the biopsies obtained after docetaxel or doxorubicin/cyclophosphamide chemotherapy suggesting that CD44^+^ cells may be intrinsically resistant to conventional chemotherapy [92].

Hong *et al.* selected pancreatic cancer cells with gemcitabine resistance. The resistant cells were more tumorigenic *in vitro* and *in vivo*, had a greater sphere forming ability and an increased fraction of CD44^+^ positive cells compared to the parental cells. Upregulation of ABCB transporters is correlated with gemcitabine drug resistance, and the expression of the ABCB1 (MDR1) transporter was increased in the resistant cells indicating a contribution of the transporter molecule for the resistance phenotype. In human pancreatic cancer samples CD44 expression was correlated with histological grade and patients with CD44 positive tumors showed poor prognosis [93]. In another study, CD133^+^CXCR4^+^ cancer stem cells derived from human pancreatic cancer tissue were highly resistant to standard chemotherapy. Moreover, the study demonstrated that tumor metastasis depended on a subpopulation of migrating CD133^+^CXCR4^+^ cancer stem cells [74].

Purified CD133^+^ cells, isolated from a human hepatocellular carcinoma (HCC) cell line and mouse xenografts were found to be more resistant to the chemotherapeutic drugs doxorubicin and fluoruracil compared to cells lacking the CD133 phenotype. The resistance phenotype correlated with the expression of anti-apoptotic proteins involved in the Akt/PKB and Bcl2 pathway, and it was therefore suggested that HCC-CSC contribute to chemoresistance through the activation of survival pathways [94]. 

CSC of colon cancer also express the cell surface marker CD133. Within the tumor, the CD133^+^ subpopulation is more resistant to chemotherapeutic drugs than differentiated cells. The resistance seems to be due to the release of IL-4 [95]. CD133^+^ cells from a human malignant melanoma cell line G3361 were found to express the human transporter ABCB5 and showed chemoresistance to doxorubicin. The increased resistance to doxorubicin is most likely a result of diminished drug accumulation due to expression of ABCB5 [96].

In a study using a number of cancer cell lines, which were stained for CSC markers and sorted, the radiosensitivity was investigated in sorted *versus* unsorted cells. While MDA-MB-231 breast CSC were found to be more radioresistant than unsorted cells, CSC of cell lines of pancreatic, colorectal cancer and glioblastoma showed no radioresistance suggesting that in permanent cancer cell lines CSC do not have a general radioresistant phenotype [97].

A number of studies investigated CSC in acute myeloid leukemia and their chemo-and radioresistance. In acute myeloid leukemia the CSC are considered to be within the CD34^+^/CD38^−^ population. In an early study, Costello *et al.* isolated CD34^+^/CD38^-^ cells from blood samples of healthy donors or AML patients. CD34^+^/CD38^-^ had an increased resistance to daunorubicin, which was connected to an increased expression of multidrug resistance genes and a lower expression of Fas/Fas-L and Fas-induced apoptosis. The resistance phenotype seems to be at least in part due to reduced drug influx and alterations in the apoptotic pathway [98]. An involvement of ABC transporters for drug resistance has also been reported for CD34^+^/CD38^−^ leukemia CSC [99]. In a separate study, bone marrow and peripheral blood cells derived from patients with AML showed a significantly higher drug efflux in the CSC compared to the non CSC representing a mechanism how the CSC could escape the effect of the cytostatic drugs [100].

## 8. Clinical Implications of CSC

**Figure 4:**
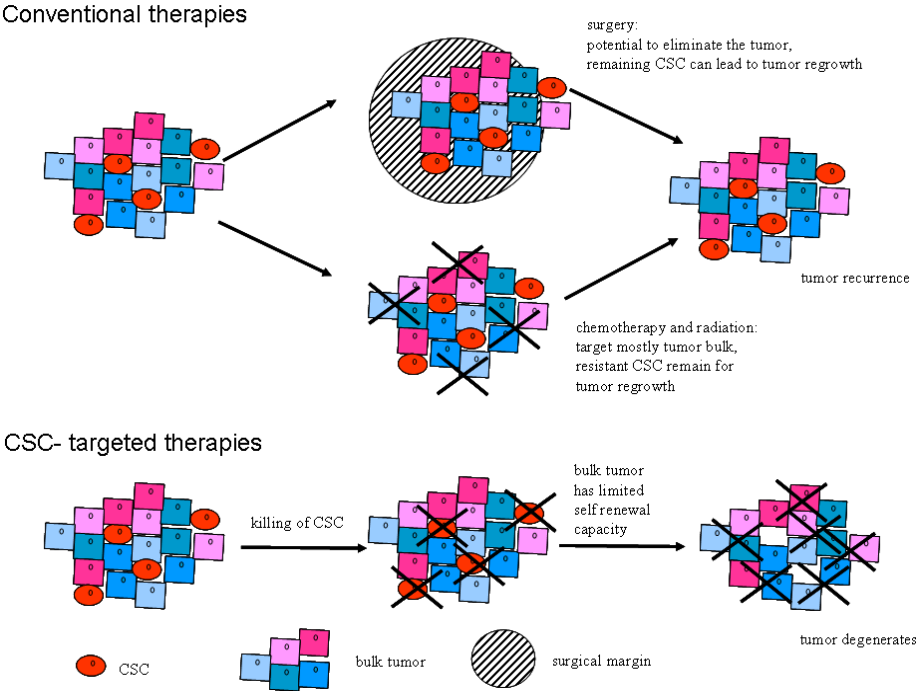
Illustration of therapeutic approaches to tumor elimination. Conventional therapies of solid tumors aim unselectively at removing the bulk tumor mass by surgery with safe surgical margins and, depending on the tumor stage, are often combined with radiation and chemotherapy. Missed CSC due to incomplete removal of the tumor or resistance to treatment will lead to tumor regrowth and ultimately failure of the therapy. Future CSC-targeted therapies may cut off the rejuvenating cell supply by CSC for the tumor and thereby lead to degeneration and involution of the tumor and lasting cure.

The current knowledge of the existence of CSC begins to lead to studies of their specific elimination (Figure 4 and Table 2). 

This could be of clinical benefit because abrogating the replenishing pool of cancer cells ultimately would stop tumor growth and lead to tumor involution as bulk tumor cells die off. This has been documented in animal experiments where removal of CSC and transplantation of only the non-CSC tumor cells did not lead to sustained tumor growth.

In addition, the evaluation of the frequency of CSC in a given tumor of a patient may be of prognostic value for the overall survival and risk of recurrence. The characteristics of a given CSC population for their marker gene expression and their proliferative state or drug resistance may be informative for the efficacy or uselessness of certain treatment options. 

The development of CSC targeted therapy has to overcome three major hindrances: (a) chemoresistance, (b) resistance to radiotherapy, and (c) immune-escape-mechanisms of CSC. 

The first two points were already addressed in Section 7 “Cancer stem cells—radiation and chemotherapy”. One very attractive approach of specifically targeting CSC is to develop antitumor T-cell vaccines. The results of these experimental therapies might have been disappointing in clinical studies for the same reasons of which established therapeutic modalities often fail: resistance of CSC. One could hypothesize that if immunotherapies could specifically target CSC, these limitations could be overcome and clinical success could be achieved. One potential target in HNSCC is the recently described CD8 defined T-cell epitope of ALDH1 [101] or the development of a CSC-dendritic cell vaccine [102]. Success of these potential therapies will depend on how well immunological responses to CSC can be modulated for example by vaccine adjuvants upregulating antigen-processing and presentation. For example, in breast cancer cells and in gliomas a reduced activity of the 26S proteasome was recently observed as a feature of CSC [40]. This may result in reduced antigen-processing and presentation of peptides presented to the immune system on major histocompatibility complex -I molecules. Therefore, CSC may be immunologically silent. Reduced proteasomal activity was also used as explanation for the high expression level of known stem cell markers like BMI-1 and nestin in CSC [40,103,104]. 

**Table 2:** Examples of targeted CSC-therapies.

Mode	Target	Tissue	Ref.
Antigen specific immunotherapy	- Dendritic cells loaded with CSC as antigen source - CD8 defined ALDH1-specific epitope	Glioblastoma, HNSCC	[101,102]
Knockdown of BMI-1 gene expression by siRNA	Bmi-1, a member of the Polycomb family of transcriptional repressors that mediate gene silencing by regulating chromatin structure. BMI-1 is essential for maintaining the self-renewal abilities of adult stem cells and CSC.	HNSCC	[105]
Combined genetic knockdown of Snail and radiochemotherapy	Snail induces EMT, which converts epithelial cells into migratory mesenchymal cells by repressing E-cadherin, desmoplakin, and cytokeratin 18, while its expression is associated with enhanced vimentin and fibronectin production.	HNSCC	[37,63,106,107]
Antibody-based target immunotherapy	- Anti-CD133 antibody-drug conjugates (ADCs) -anti-interleukin-3 (IL-3) receptor alpha chain (CD123)-neutralizing antibody - antiCD44a6 - Anti-ABCB5	- HNSCC hepatocellular and gastric cancers - AML in a SCID mouse model - melanoma	[37,108,109]
Modulation of CSC differentiation	Bone morphogenetic proteins (BMPs) induced differentiation of CD133^+^ brain tumor stem cells, weakening their tumor-forming ability.	glioblastoma	[110]

A second immunotherapeutic approach to target HNSCC is the use of monoclonal antibodies. Three different strategies have thus far entered the clinic: (a) Antibodies directed against tumor surface antigens that trigger immune effector cells that cause tumor cell death; (b) antibodies that are conjugated to cytotoxins or radiation emitters causing cell damage directly upon binding; and (c) antibodies blocking or inhibiting cellular pathways after binding to the respective receptor. These strategies have resulted in various degrees of improved prognosis and survival, but not yet in cure. This variable success can be explained with tumor immune-escape (e.g., downregulation of the target) and a heterogeneous expression of the antibody targets in the tumor. A number of studies investigating the use of antibodies targeting CSC of solid cancers are underway [37,111]. 

To date, no antibody selectively targeting CSC in HNSCC has been described, however candidates are under investigation. Currently a chimeric human/murine monoclonal anti-EGF-R antibody (Cetuximab) is in clinical use that showed in preclinical studies three different mechanisms affecting tumor cells: (a) enhanced tumor cell apoptosis, inhibition of proliferation and invasiveness by blocking the tyrosine-kinase mediated pathways; (b) antibody-dependant cell mediated toxicity; and (c) blockage of the nuclear import of EGF-R preventing activation of DNA repair. A specific affinity of these antibodies towards CSC has not been described. *In vitro* testing, however, showed that activation of EGF-R in HNSCC leads to an increased side population (SP) as defined by HOECHST dye, and conversely, inhibition of EGF-R leads to a decrease in SP implicating a possible role of EGF-R in regulating HNSCC-CSC [112]. CD44v6 antibodies either radiolabeled or coupled with a cytotoxic drug entered phase I clinical testing in patients suffering from HNSCC. As discussed before, the role of CD44 to identify CSC in head and neck cancer remains controversial. Nevertheless in a phase I dose escalation study the treatment showed promising anti-tumor effects. One patient however developed toxic epidermal necrolysis and died, indicating that perhaps anti-CD44v6 was not exclusively targeting the CSC or even bulk tumor respectively. This observation may be supported by immunohistological studies showing that the expression of CD44v6 is not exclusively restricted to the tumor [64]. 

Whether single antibody treatment will be effective in eliminating CSC or if combining different antibodies that aim at separate CSC targets will be necessary for successful elimination of CSC remains unresolved until they become available to clinical testing. Nevertheless, since antibody treatments show a toxicity profile different from cytotoxic agents or radiotherapy they can be combined with these treatment modalities and may therefore provide an additional treatment option in the future.

## 9. Conclusions

Initiation of malignant tumor growth depends on the transformation of somatic cells leading to the acquisition of abilities like immortality, self-renewal, and a de-differentiated phenotype like the ones normal stem cells have. The concept is that either a somatic stem cell is being transformed and gives rise to tumor cell heterogeneity, or a tumor cell can dedifferentiate and become a cell with stem cell-like features. There is some evidence arguing for the CSC model describing the monoclonal origin from a stem cell and development of heterogeneity during growth of the tumor mass.

The detection of CSC in certain malignancies has fostered studies to identify and characterize equivalent cells in many tumor entities. Several methods have been developed that are useful to describe the characteristics of these cells. It turned out that such cells can be found but to date the markers available to identify CSC remain rather specific for the tissue of origin than for the CSC per se. A generic marker has not been found so far. 

Due to their high tumorigenicity and drug resistance, CSC are thought to be responsible for tumor regeneration after chemotherapy. We can speculate that a tumor with a high proportion of cancer stem cells may be associated with resistance to chemotherapy and that the proportion of stem cells may increase due to positive selection after chemotherapy because of their resistance. This may lead to a more aggressive tumor-phenotype, a hypothesis that is supported by clinical observation. 

In head and neck cancer as in other malignancies, the presence of cancer initiating and sustaining cells can be postulated. Initial experimental results demonstrate that CSC-like cells can be isolated and further characterized from HNSCC tumors and cell lines. At present, a definite marker for HNSCC stem cells has not been described but markers that can be used to enrich for tumor cells with CSC-properties have been identified.

Ultimately, the identification of conclusive CSC markers and definition of the impact on therapeutic outcome may lead to diagnostic procedures for evaluation of CSC content and adequate therapeutic strategies. Furthermore, specific CSC markers could also serve as potential targets for upcoming therapies.
